# The objective structured clinical examination as an assessment strategy for clinical competence in novice nursing practitioners in Taiwan

**DOI:** 10.1186/s12912-021-00608-0

**Published:** 2021-06-07

**Authors:** Sue-Hsien Chen, Shu-Ching Chen, Yo-Ping Lai, Pin-Hsuan Chen, Kun-Yun Yeh

**Affiliations:** 1 Chang Gung Medical Education Research Centre, Chang Gung Memorial Hospital, Linkou, Taiwan; 2 Department of Nursing Management, Chang Gung Medical Foundation Administration, Taoyuan, Taiwan; 3 School of Nursing, Chang Gung University, Taoyuan, Taiwan; 4 Department of Medical Research, National Taiwan University Hospital, Taipei, Taiwan; 5 Department of Internal Medicine, National Taiwan University Hospital, Taipei, Taiwan; 6 Division of Hemato-oncology, Department of Internal Medicine, College of Medicine, Chang Gung Memorial Hospital, Keelung & Chang Gung University, 222 Maijin Road, Keelung, Taiwan

**Keywords:** clinical competence, new nurses, occupational stress, objective structured clinical exam (OSCE)

## Abstract

**Background:**

The conventional written tests and professional assessment have limitation in fair judgement of clinical competence. Because the examiners may not have total objectivity and may lack standardization throughout the assessment process. We sought to design a valid method of competence assessment in medical and nursing specialties. This work was aimed to develop an Objective Structured Clinical Exam (OSCE) to evaluate novice nursing practitioners’ clinical competency, work stress, professional confidence, and career satisfaction.

**Methods:**

A Quasi-experimental study (pre-post). Fifty-five novice nursing practitioners received the OSCE three-months following their graduation, which consisted of four stations: history taking, physical examination, problem-directed management, interpersonal communication, and the required techniques of related procedures. The examiners had to complete an assessment checklist, and the participants had to complete a pre-post questionnaire (modified from a Nursing Competency Questionnaire, a Stress scale, and Satisfaction with Learning scale).

**Results:**

Among the novice nursing practitioners, 41 of them (74.5 %) passed the exam with a mean score of 61.38 ± 8.34. There was a significantly higher passing rate among nurses who were working in medical-surgical wards (85.7 %) and the intensive care unit-emergency department (77.8 %) compared to novice nursing practitioners working in other units. All the novice nursing practitioners at Station A had poor performance in assessing patients with a fever. OSCE performance was more associated with educational attainment and work unit, rather than the gender. Finally, the participants showed statistically significant increases in their clinical competency, confidence in their professional competence, satisfaction with the clinical practice, and decreased work stress after the OSCE.

**Conclusions:**

We found that the OSCE process had a positive educational effect, in providing a meaningful and accurate assessment of the competence of novice nursing practitioners. An appropriate OSCE program is vital for novice nursing practitioners, educators, and administrators. The effective application of OSCEs can help novice nursing practitioners gain confidence in their clinical skills.

## Background

Nursing competency is considered as an integrative ability of clinical knowledge, judgment, skills, attitude, and beliefs to perform specific practice settings in different situations [1, 2]. Competence also reflects the holistic nursing care. Poor nursing competence decreases the quality of care and patient safety [3, 4]. Novice nursing practitioners (NNPs) usually have difficulty practicing competently, which can compromise their quality of patient care [5, 6]. The transition period to achieving competency can be a time of strain for NNPs [7]. Thus, the turnover rate of nurses tends to be high at the start of their careers. Additionally, patients are increasingly interested in customized treatment. Inadequate nursing competency may affect implementation of multidisciplinary team plan. Such issues may be resolved by offering NNPs an appropriate program of competency assessment and in-service education that may improve their competence and help them adapt to their work environment.

Conventional assessment methods used for clinical competence remains a matter of concern because the examiners may not have full objectivity and standardization through the assessment process [8]. Furthermore, it is risky for healthcare institutions to have NNPs demonstrate their ability with real patients and clinical situations during their training period, even though this type of assessment may yield reliable results that reflect the true competence of NNPs. Therefore, appropriate assessment of competence remains an ongoing challenge for responsible institutions in training NNPs. The Objective Structured Clinical Evaluation (OSCE), a multidimensional practical examination of clinical performance, is able to reflect problem-solving abilities, critical thinking and communication skills of healthcare professionals [8, 9] and has been reported to be a feasible method of assessing the competence in undergraduate/postgraduate medical education, paramedical-specialist training, and licensing examinations [8, 10, 11]. It provides for a meaningful alternative strategy as it allows for individual assessments of a total group in a timely, controlled and safe way. Recently, the application of the OSCE to assess clinical skill competency has gained attention in nursing education [12]. The OSCE station content varies according to student experience and the nature of the assessment. The types of problems portrayed in an OSCE are those that students would commonly encounter in a clinic or hospital [12]. Throughout the OSCE, examinees show their clinical competence in a safe clinical scenario and educators can audit the weak or missing competencies of examinees [13, 14]. Hence, OSCE is an assessment that allows examinees to demonstrate their nursing competence in a simulated clinical setting that reflects the clinical competence that nurses need to care for patients [15–18].

Although using the OSCE to assess clinical competence of NNPs might not truly reflect how nurses will perform in the clinical setting, it remains an important strategy as it falls just short of the optimal practice-based assessment and above the use of written assignments or multiple-choice question. Based on the previous researches, the aim of the study is to investigate the impact of an OSCE program on the learning progression of NNPs.

## Methods

### OSCE setting and participants

**Fig. 1:**
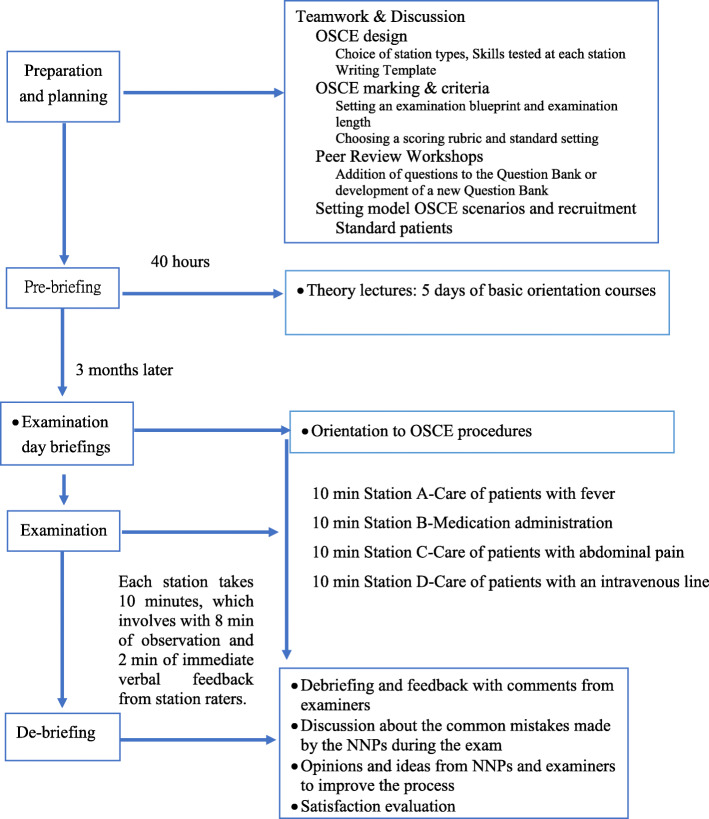
The diagram illustrates the steps necessary to perform OSCE for novice nursing practitioners

We conducted the examination three months after the NNPs started their careers at our institution. We tested their clinical skills related to the issues addressed at each station: history taking, conducting a complete physical examination, problem-directed management, interpersonal communication, and required-procedure techniques (Table 1). We created a 4-station OSCE: (1) Care of fever (Station A), (2) medication administration (Station B), (3) patients with abdominal pain (Station C), and (4) care for intravenous lines (Station D) (Fig. 1).

**Table 1:** Description of the four OSCE^a^ stations for assessing the competence of novice nursing practitioners

*Station*	*Description of competence*	*Task*	*Skills tested*
A	Care of patients with fevers	Evaluate performance of history taking and symptom assessment	1. History taking: medical history, drug history, travel history, and symptoms 2. Explain the procedure for fever management and its purpose. 3. Answer questions and provide emotional support. 4. Education, including home care
B	Medication administration	Evaluate performance of medication administration and patient education	1. Perform 5 ‘Rights’: right drug, right route, right time, right dose, and right patient. 2. Know medication-associated risks. 3. Know the effects: primarily intended effect, and related effects of the pharmacological properties. 4. Communicate clearly. 5. Monitor adverse effects. 6. Report errors and adverse events.
C	Care of patients with abdominal pain	Evaluate performance of history taking, physical examination and team work	1. Perform comprehensive assessment of abdominal pain, including location, characteristics, onset, duration, frequency, quality, intensity, severity, precipitating, and relief factors. 2. Communicate with the interdisciplinary team.
D	Care of patients with IV^b^ lines	Observe administration of IV medication and monitoring of the line to ensure that it is working without complications	1. TOUCH: check if there is a temperature change (heat or warmth), redness, or leakage at the IV site. 2. LOOK: confirm that the IV site is dry and visible at all time. 3. COMPARE: check if there is swelling in the limb with the IV line, comparing with the opposite limb without the IV line. 4. Educate: provide information about IV care to patients and caregivers.

^a^ OSCE, objective structured clinical exam; ^b^ IV, intravenous

We chose these four clinical scenarios because nursing staff with over 10 years of clinical experience at our institution reviewed the relevant literature [3, 19–22] and recommended that the proper handling of these clinical problems can be essential for NNPs at the beginning of their careers. Furthermore, over 90 % of the experts on the OSCE education committee of our institution agreed on the importance and practicality of each clinical scenario. The internal consistency of the OSCE stations was tested using Cronbach’s alpha. The overall reliability of the Cronbach’s α coefficient was 0.791, which indicated good stability and internal consistency, with minor differences in the progression of the indices.

This comprehensive 4-station OSCE was carried out at Chang Gung Memorial Hospital, Keelung, Taiwan between August 2017 and July 2018. The population of the research consisted of the fifty-five NNPs from different work units, who obtained a diploma of bachelor’s degree in Nursing in Taiwan, but had no internship experience in clinical practice as of July 2017. None of the NNPs had ever taken part in an OSCE before this study. All 55 participants completed the following required test items and questionnaires at the end of this study. Before entering the OSCE, NNPs needed to complete the training of core professional skills, a 5-day orientation that included standard training courses that were designed and verified by the Department of Nursing at our institution [3]. Three months after the orientation courses, the NNPs were assessed at end of the module through formative OSCE. The NNPs were familiarized with the OSCE procedure under the guidance of instructors who were nursing staff at our institution with comprehensive training. The instructors encouraged the NNPs to discuss the core and provide feedback to the NNPs about his/her achievements, deficiencies and opportunities for improvement.

### Implementation, instruments, and evaluations at the OSCE stations

Each station had one standardized patient (SP) and one examiner. The SP was a person who had completed at least eight hours of standard training provided by the Taiwan Association of Standardized Patients, who was also capable of simulating the signs and symptoms of diseases, mimicking clinical scenarios, and providing feedback to the NNPs. The examiner was a nursing faculty rater who had completed OSCE education training program and was certified by our institution and the Taiwan Nursing Association. The raters acted as passive evaluators and were instructed not to guide or prompt the participants.

**Fig. 2:**
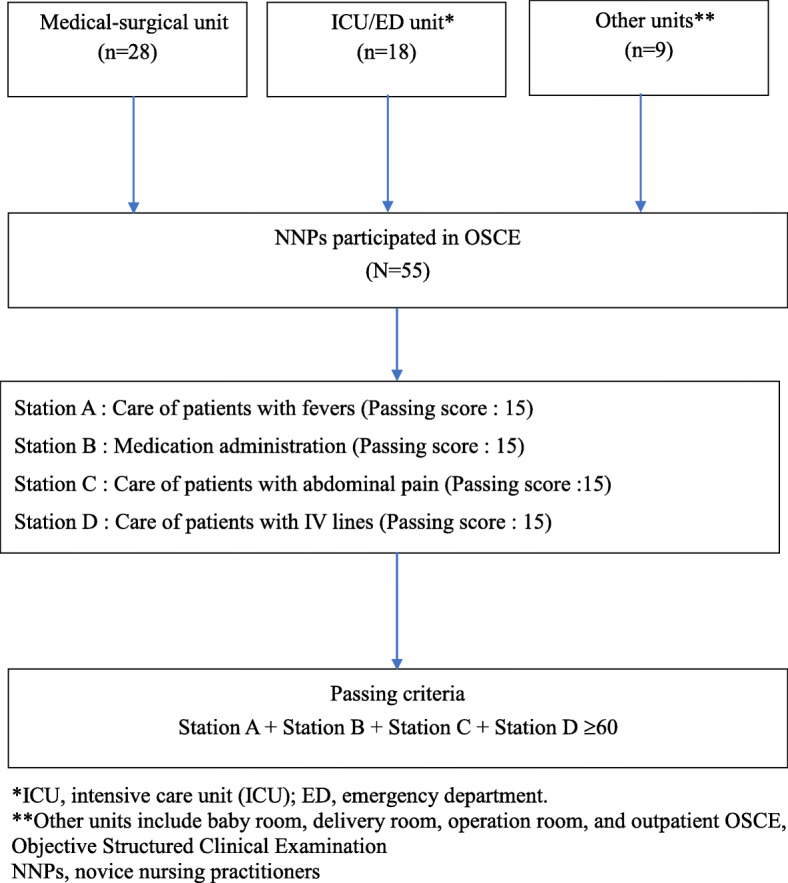
Flow chart of enrollment and evaluation of NNPs in OSCE

At the beginning of the test at each OSCE station, participants had 1 min to read a written description of the required tasks. Participants would spend 10 min at each station, consisting of 8 min of observation and 2 min of immediate verbal feedback from the station examiner (Fig. 1). The examiner would assess the abilities of the participants in terms of their clinical skills, strategies, and interpretation of clinical problems (Table 1), and grade them according to a checklist for each skill. The checklist consisted of 10–12 items that were rated on a 3-point scale: 0 (failed to perform), 1 (performed poorly or out of sequence), and 2 (performed appropriately in the correct sequence). Kendall’s coefficient of concordance was 0.781 (*p* < 0.0001), indicating that there was a significant correlation between the examiners’ scores; consequently, there was a good agreement between examiners’ and the scorers’ ratings. We also measured certain practices, such as greeting the patient and hand decontamination, but we did not apply these elements to participants’ overall scores. We recorded the sum of the scores from all the checklist items for each station, and the participants received their own performance-analysis report after the OSCE (Fig. 2). The instructors arranged an 80-min debriefing session to review the report and help the NNPs understand the core (i.e. clinically important) elements of the stations. We used the “borderline-group method” to establish the standard “pass” score. The “pass” score was the mean score of the NNPs whose OSCE scores were rated “borderline” at each station [22].

Participants were required to complete a questionnaire before the implementation (pretest) and after the end of the OSCE program (posttest). The questionnaire was a modified version of a tool used in a previous report, which collected basic learning and personal background information, plus a Nursing Competency Questionnaire (NCQ), a Stress scale, and a Satisfaction with Learning scale [3]. The Nursing Competency Questionnaire, a 26-item instrument using a five-point scale, was designed to evaluate nursing competency. The five domains include taking a medical history (5 questions), physical assessment (3 questions), interpersonal communication (7 questions), problem-directed management (5 questions), and problem-required skills (6 questions). The Stress scale consisted of 10 statements in relation to stressful nursing situations. Each item required respondents to rate the situation on a 5-point scale (1 = not stressful at all to 5 = extremely stressful). The Satisfaction with Learning scale, a 3-item instrument designed to measure the nurses’ satisfaction. Each item contained a statement about nurses’ satisfaction with learning in regards with obtaining input from trainers, using a 5-point scale from 1 (strongly disagree) to 5 (strongly agree). Seven experts, including three attending physicians and four senior nursing supervisors, were invited to validate this questionnaire. A test of internal reliability was conducted with ten senior nurses who had more than 5 years of working experience. Next, the professionals rated its content validity, which yielded a content validity index (CVI) of 0.89–0.91.

### Ethical considerations

Data collection began after the research ethics committee had (IRB approval number:104-9928B) approved the study protocol at the host hospital. Subsequently, we held a meeting with the NNPs to explain the program and the study, including the study’s purpose and procedures, the participants’ rights, and confidentiality. We sent this information, including a covering letter and the questionnaire, to the participants before data collection in a self-addressed stamped envelope. For their convenience, the participants could complete the questionnaires in either the paper or electronic form. The participants returned the questionnaires by mail or emailed them to the research team. We destroyed all the envelopes and deleted all the email addresses that could identify the participants, immediately after the data were saved in a secured computer protected with passwords known only to the primary investigator.

### Data analysis

The data were verified and analyzed using the Statistical Package for Social Sciences (SPSS) software, version 21.0 for Windows. Descriptive statistics (mean scores and standard deviations) were obtained for each examination tool, and analyzed by one-sample or two-sample *t-*tests, or analysis of variance when appropriate. Statistical tables and percentages were used for the presentation of demographic data; the chi-square test and Spearman’s correlation were used to test the significance of associations between demographic variables and competency levels. Continuous data were tested for normality using the Kolmogorov-Smirnov test and presented as means and standard deviations. The internal consistency of the OSCE stations was tested using Cronbach’s alpha. Agreement between the total scores obtained in both tests was analyzed using the Bland-Altman analysis, and associations were measured using Pearson’s correlation coefficient. The level of significance for all analyses was set at 5 % (*p* < 0.05).

## Results

### Demographic features of the participants

The characteristics of the participants are shown in Table 2. The examinees came from four different units, they were analyzed by different variables, including gender, educational level, and experiences of previous OSCE. From the 55 participants, 50 were female and 5 were male; the age of all the participants ranged from 20 to 29 years old. The majority of the examinees had graduated from college (74.5 %, *n* = 41) and half of them worked in medical or surgical wards (50.9 %, *n* = 28).

**Table 2:** Demographic characteristics of the study participants (*N* = 55)

Characteristics	No (%)
Age, Mean ± SD	23.38 ± 1.86
Gender	
Male	5 (9.1 %)
Female	50 (90.9 %)
Educational level	
Junior college	14 (25.5 %)
College and above	41 (74.5 %)
Unit	
Medical-surgical wards	28 (50.9 %)
Intensive care unit	18 (32.7 %)
Other (outpatient clinic)	9 (16.4 %)

### NNPs’ evaluation of the OSCE

The results were analyzed using Modified Angoff Method [23]. The passing score of each station and the passing criteria of the OSCE were shown in Fig. 2. We found that 42 NNPs (76.4 %) passed the OSCE with a mean score of 64.62 ± 5.79 (range, 56–79), whereas 13 (23.6 %) participants failed the competency test with a mean score of 48.54 ± 6.33 (range, 37–57). The participants who worked in “Other” units (55.6 %) had a significantly higher failure rate than participants who worked in medical-surgical wards (17.9 %) and the intensive care unit-emergency department (16.7 %, *p* < 0.05). Table 3 shows that the OSCE performance was associated with educational attainment, gender, and work unit. Regarding the gender difference, the male participants (67.40 ± 8.26) performed better than female ones in general(60.30 ± 8.93; *p* = 0.009). Overall, the performance of participants in college and above (62.41 ± 8.44) was better than that of Junior college (56.64 ± 9.67; *p* = 0.038). In concern with the unit difference in station C, the best performance units were critical care and emergency units, followed by medical-surgical and other units (16.33 ± 3.48 vs. 15.32 ± 3.61, 12.44 ± 2.79, *p* = 0.028). Overall, the average score of participants in the medication administration station (Station B) was higher than that of the other 3 stations. Fewer participants had relatively low pass rates in Stations A and C, particularly for the patient with a fever in station A, of which the NNPs had the lowest average scores (10.42 ± 3 0.00) and the fewest pass rates (10.9 %) (Table 3).

**Table 3:** Summary of the OSCE scores in the entire group and subgroups, stratified by gender, educational level, and work unit

Stations Assessment	No.	Overall	*p*	Station A	*p*	Station B	*p*	Station C	*p*	Station D	*p*
**Score (items)**	55	96(48)		24(12)		24(12)		24(12)		24(12)	
**Mean ± SD**		60.82 ± 9.04		10.42 ± 3.00		17.42 ± 3.42		15.18 ± 3.63		17.80 ± 4.68	
**Range**		37–79		3–16		9–23		6–22		4–24	
**Passing score**	55	58		15		15		15		15	
Number of fail		13		49		7		24		7	
Pass rate (%)		76.4		10.9		87.3		56.4		87.3	
**Gender**			0.048		0.43		0.001		0.99		0.44
Male	5	68.40 ± 7.86		9.40 ± 4.62		22.00 ± 1.22		15.20 ± 4.55		21.80 ± 2.68	
Female	50	60.06 ± 8.87		10.52 ± 2.85		16.96 ± 3.22		15.18 ± 3.58		17.40 ± 4.67	
**Educational level**			0.041		0.19		0.73		0.12		0.11
Junior college	14	56.57 ± 9.82		9.50 ± 3.01		17.14 ± 4.02		13.86 ± 3.21		16.07 ± 4.68	
College and above	41	62.27 ± 8.41		10.73 ± 2.98		17.51 ± 3.24		15.63 ± 3.69		18.39 ± 4.59	
**Unit**			0.015		0.43		0.37		0.028		0.123
Medical-surgical	28	62.07 ± 7.57		10.93 ± 2.64		17.54 ± 3.00		15.32 ± 3.61		18.25 ± 3.93	
ICU	18	62.77 ± 9.38		10.00 ± 3.27		17.94 ± 3.22		16.33 ± 3.48		18.50 ± 4.49	
Other	9	53.00 ± 9.46		9.67 ± 3.57		16.00 ± 4.50		12.44 ± 2.79		14.89 ± 4.68	

OSCE, objective structured clinical examination; Station A, care of patients with fevers; Station B, medication administration; Station C, care of patients with pain; Station D, care of patients with intravenous lines

### Clinical competence of novice nursing practitioners

Shown in Table 4, the participants had statistically significant increases in their clinical competency, their confidence in professional competence, their satisfaction with clinical practice, and a significant decrease in work stress after taking the OSCE. We found that 14 NNPs who failed this test, gained greater confidence in their competence (10.40 ± 0.1.53 vs. 12.54 ± 0.1.92, *p* = 0.044) and had greater satisfaction (9.75 ± 0.0.50 vs. 12.29 ± 0.1.54, *p* = 0.042) after taking the OSCE. No significant differences in confidence, competence, work stress, or satisfaction were found among the nurses who worked in different units (data not shown). Nevertheless, the NNPs working in other units had significantly (*p* = 0.032) lower scores on clinical competence (58.32 ± 6.32) than NNPs working in the medical-surgical wards (62.75 ± 3.53) and the intensive care unit-emergency department (61.36 ± 2.89).

**Table 4:** Changes in nursing competency, stress, confidence in professional competence, and nurses’ satisfaction between the beginning and end of the study^a^

Variables are expressed as mean score ± standard deviation	Initial-OSCE	Post-OSCE	*p*
Competency	57.44 ± 5.91	61.38 ± 8.34	0.001
Medical history taking	11.75 ± 3.16	11.84 ± 3.11	
Complete physical examination	9.53 ± 2.87	9.71 ± 2.91	
Interpersonal communication,	5.47 ± 1.37	5.47 ± 1.32	
Problem-directed management	9.45 ± 3.35	12.20 ± 3.86	
Required procedure skills	21.24 ± 4.32	22.16 ± 4.21	
Stress	26.76 ± 7.63	25.60 ± 4.42	0.002
Confidence in professional competence	11.78 ± 1.99	13.00 ± 2.70	0.013
Nurses’ satisfaction	11.95 ± 2.47	13.04 ± 3.20	< 0.001

^a^ Initial-OSCE, beginning of the study; Post-OSCE, end of the study

## Discussion

The current study shows that OSCE delivers a practical strategy for nursing educators and healthcare administrators to improve the clinical ability of NNPs. Through multiple feedback sessions and debriefing, teaching faculty can understand what types of clinical abilities NNPs need to improve in their practice. During the OSCE in our study, the faculty obtained immediate feedback from the NNPs, and could assess the suitability of the current clinical teaching program for the NNPs’ learning. Although preparation for conducting an OSCE is time-consuming and requires careful planning, this teaching program offers valuable assistance in evaluating clinical performance. We found that an OSCE program for NNPs improved clinical competency and reduced work-related stress. In addition, the OSCE helped increase the NNPs’ confidence and reduce their personal embarrassment when encountering similar clinical situations. Previous reports showed that nursing students found that the OSCE help them deal with stressful clinical situations and develop their confidence in clinical practice [24–27]. Student midwives saw the OSCE as a valid means of assessment and that it increased their confidence in performing clinical skills [26, 28]. These findings support our results that OSCE program is an appropriate method for accurately measuring and effectively addressing weaknesses, in order to improve the competence of NNPs in daily practice.

Some interesting observations and study concerns can lead to further discussion. First, previous research suggested there was a positive correlation between the number of stations and reliability [10, 29]. Four stations were included in this OSCE based upon clinical experts’ experience at our institution. The overall reliability in the present study was 0.791, which is a desirable level of reliability for high-stakes tests, such as certification [30–32]. The number of stations in this OSCE was, thus, appropriate to assess the competence of the NNPs in our study. Second, the lowest OSCE score and number of NNPs passing the station tests occurred in Station A- care for fever. Also, participants from other units (e.g., the baby room, delivery room, and operation rooms) performed not well in overall tests and Station C-care for abdominal pain. Through the debriefing session, we found that the NNPs were unable to perform well in history taking and symptom assessment related to the patient’s problems. These results may be attributed to the fact that they had few opportunities to encounter patients with these clinical scenarios during the first three months of their careers. To improve the clinical ability of NNPs who failed, we arranged interactive teaching sessions in work units. Senior instructors with over 5 years of clinical experience provided opportunities for NNPs to work with patients with clinical problems and guided them in interacting with the patients, including, questioning patients and reflecting on what they had learned. The instructors also focused on teaching NNPs how to recognize the signs and symptoms, understand daily medication regimens, interpret abnormal laboratory data, and facilitate communication between the nurses and patients. Likewise, since all 5 male-gender NNPs in this study came from either medical-surgical wards or the intensive care unit-emergency department, they had a better chance to encounter medical events presented at OSCE stations and consequently gained better score performance. Lastly, Saito et al. reported that adoption of the OSCE in medical education is effective for the training of medical students by developing necessary basic skills in both technical and behavioral aspects, and it enables educators to guide students toward the appropriate integration of knowledge, skills, and behavior [33]. Even though some NNPs failed the OSCE, they still benefited from it in terms of confidence in clinical competence and satisfaction with OSCE learning in our study. The feedback section following assessment of performance of students is a vital element in their learning process[34]. We believe that it is most effective to improve competence if given immediately after examination. The OSCE itself provided NNPs with a bi-directional feedback mechanism to measure their strengths and weaknesses in clinical skills.

The major limitation of this study lies on the fact that it did not compare the OSCE to the conventional methods of assessing the competence of NNPs. However, the OSCE supplanted the direct observation of actual patients and offered a sound assessment of competence and improvements among NNPs in the current study. And the OSCE offers an objective and standard tool to assess multifaceted clinical ability of NNPs in a close-to-clinic situation. Also, it is an appropriate method for accurately measuring and effectively addressing the weaknesses, in order to improve the competence of NNPs in daily practice.

Taken together, our results further support the notion that OSCE training, with efficient interactive communication, is mutually beneficial to the NNPs and the training staff involved in the learning process. This educational approach requires robust design based on sound pedagogy to assure practice and assessment of holistic nursing care.

## Conclusions

A well-designed OSCE has a positive educational impact, offer an appropriate professional assessment, and helps NNPs gain confidence and improve their clinical competence. We believe that OSCE is an effective and authentic mode of assessment and can be applied to other levels of nurses as well.

## Data Availability

The datasets generated and/or analyzed during the current study are available from the corresponding author on reasonable request.

## Abbreviations

ICU: Intensive Care Unit
IV: intravenous
NNPs: Novice nursing practitioners
NCQ: Nursing Competency Questionnaire
OSCE: Objective Structured Clinical Exam
SD: Standard Deviation
